# Asian Indians With Prediabetes Have Similar Skeletal Muscle Mass and Function to Those With Type 2 Diabetes

**DOI:** 10.3389/fnut.2019.00179

**Published:** 2019-11-27

**Authors:** Sucharita Sambashivaiah, Stephen D. R. Harridge, Nidhi Sharma, Sumithra Selvam, Priyanka Rohatgi, Anura V. Kurpad

**Affiliations:** ^1^Department of Physiology, St. John's Medical College, Bengaluru, India; ^2^Centre for Human and Applied Physiological Sciences, King's College London, London, United Kingdom; ^3^Division of Nutrition, St. John's Research Institute, Bengaluru, India; ^4^Division of Epidemiology and Biostatistics, St. John's Research Institute, Bengaluru, India; ^5^Apollo Hospitals, Bengaluru, India

**Keywords:** skeletal muscle, muscle mass, muscle function, body fat, diabetes mellitus, prediabetes

## Abstract

**Background:** Type 2 Diabetes (T2D) is a major concern among Asian Indians, not least because many develop T2D at despite having a normal BMI (body mass index), and with relatively low body fat. Asian Indians are also generally considered to have relatively low skeletal muscle mass and strength, this has not been explored in the context of T2D.

**Aim:** The present study aimed to compare skeletal muscle mass, function and contractile quality (strength/mass) between healthy controls, those with prediabetes (PD) as well as T2D middle-aged non-obese Asian Indians.

**Methods:** Adult males between the age of 20–50 years, consisting of healthy controls (*n* = 44), PD (*n* = 125) and T2D (*n* = 55) were studied. Skeletal muscle mass was measured using Dual X-ray Absorptiometry (DXA). Isometric and dynamic muscle function was measured using an isokinetic dynamometer (at 0, 60, 120, 180 degree/s). Muscle contractile quality was derived by dividing the peak muscle torque with the respective LMM (lower limb muscle mass). Fasting blood glucose (FBG) and insulin were used to derive insulin resistance (HOMA-IR).

**Results:** The control group was on average 10 years younger than the other two groups (*p* < 0.01). The LMM was similar across the three study groups. However, the age-adjusted mean muscle torque was significantly lower in both absolute and normalized isometric and isokinetic strength in PD and T2D groups compared to controls (*p* ≤ 0.01), with the difference persisting even after adjusting for age and other covariates. However, there was no difference in muscle strength and contractile quality between the PD and T2D study groups.

**Conclusions:** Muscle strength and contractile quality would appear to be sensitive and early indices of the trajectory toward diabetes in Asian Indians and more so than skeletal muscle mass. It is thus important to recognize the importance of functional measurements among this population when considering the role of muscle in diabetes. The data also would suggest that specific muscle conditioning (e.g., resistance training) might have efficacy in improving function as well as muscle mass, and thus aiding in the prevention of the trajectory toward the development of T2D.

## Introduction

Both contractile and metabolic signals play integral roles in regulating muscle homeostasis (1). The interrelationships between metabolic and mechanical signals help maintain contractile function (strength), metabolic health, physical well-being, and quality of life (2). Ideally, muscle bulk (mass) along with function should reflect an optimum balance between metabolic and contractile function (3). Therefore, skeletal muscle mass alone has been used as a reflection of nutritional and physical activity status in both health and disease (4). However, muscle mass is a static measure, depending on its composition of contractile and cytoskeletal proteins, as well as intra and intermyocellular fat (5). Recent evidence suggests that there is excessive intramyocellular fat in Indians with PD (6), and therefore it is likely that muscle mass may not be altered in such subjects. In such circumstances, it is likely that skeletal muscle contractile function may provide more sensitive diagnostic information, particularly in chronic disease. An example of this is the finding of a relation between all-cause mortality or morbidity and reduced forearm muscle strength (7).

A further dimension of muscle function is contractile quality. Whilst this reflects force per unit area or mass, it can also be considered relevant to an array of muscle characteristics, including glucose metabolism, intramuscular adipose tissue, capillary density, structural composition, and fatigability (8, 9). Currently, there is a lack of literature exploring the role of skeletal muscle mass, function, and contractile quality in chronic disease. This is important, since, for example, muscle function, or greater muscle contractility, has been linked to upregulation of glucose transporters (10). Therefore, it is critical to evaluate muscle function, skeletal muscle mass as well as contractile quality in populations who are considered to have low muscle mass (10), and who have a predisposition for T2D (11).

T2D is now a major concern among Asian Indians, particularly in the subcontinent, where the number has increased markedly from 26 million in 1990 to 65 million in 2016, with an increase in crude prevalence from 5.5 to 7.7% in the same time period (12). Increasing modernization and adoption of a more sedentary lifestyle that not only results in a positive energy balance and fat deposition, but also less skeletal muscle use, could contribute to the marked increase in T2D among Asian Indians (13). This occurs at a normal BMI and has led to the terms “metabolic obesity” and “the thin-fat Indian” (13). While age-related loss in muscle mass and function among T2D has been studied extensively in western populations (14, 15), there are no studies in young and middle-aged Asian Indians who constitute a major proportion of the population in India. There are also no studies that compare skeletal muscle mass and function in healthy Asian Indians with those with PD and T2D. The aim of the present study was thus to compare skeletal muscle mass, function and contractile quality of the major limb muscle (quadriceps) between healthy males, PD and T2D middle-aged non-obese Asian Indians.

## Materials and Methods

### Participants

The study population consisted of healthy controls (*n* = 44), prediabetes (*n* = 125) and T2D (*n* = 55). Adult males between the age of 20–50 years were recruited in and around St. John's Medical College and Hospital and through advertisements. The participants were reviewed for inclusion criteria at the first point of contact including their preexisting medical records. All participants were having either fasting blood sugar or HbA1C data already available at the first point of contact. This information was used to shortlist the eligible candidates. They were then invited to undergo a 75-g oral glucose tolerance test (OGTT) and HbA1C. The eligible participants were investigated for plasma glucose obtained by the GOD POD method (Beckman Coulter AU480, Japan), and glycosylated hemoglobin (HbA1c, HPLC, BioRad, Variant Turbo II, India). The previous day of the OGTT clear instructions were given to the participant to fast for 8–10 h before the testing. The American Diabetes Association Expert Committee criteria (16) were used for the diagnosis of PD. For the control group, the following criteria were used FBG, ≤ 100 mg/dL, postprandial plasma glucose, ≤ 140 mg/dL, or HbA1c ≤ 5.6%. If dysglycemia was detected by either fasting pl glucose (101–125 mg/dL), postprandial plasma glucose (141–199 mg/dL) or HbA1c (5.7–6.4%) the subject was characterized as PD. For T2D, those diagnosed earlier, with duration of <5 years, and on treatment with oral hypoglycaemic agents were recruited. The purpose of the study and the potential risks involved were explained to each subject and written informed consent was obtained. All were in good health as determined by medical history and physical examination. Those with conditions such as anemia, joint injuries, hypertension, cardiovascular disease, tuberculosis, cancer, and thyroid disorders were excluded. All participants underwent serum lipid profiling, including serum cholesterol, high-density lipoprotein (HDL), low-density lipoprotein (LDL) and triglyceride estimations using a Chemiluminescence Immunoassay (Siemens, Model EXL with LM 1 & 2, Germany). Plasma insulin was measured by electrochemiluminescence (ADVIA Centaur CP, Siemen's Healthineers, India). Insulin resistance and beta-cell function (HOMA-IR and HOMA-%B) were assessed by the homeostatic method using standard formulae for calculation (17). Physical activity levels (PAL) were calculated based on a physical activity questionnaire (18). All protocols were approved by the Institutional Ethics Committee, St. John's National Academy of Health Sciences, Bengaluru, India (IEC reference number 66-2014/112-2014 /116-2015).

### Body Composition

All participants underwent anthropometric assessment. This included weight recorded in minimal clothing to the nearest 0.1 kg, using a digital scale (Salter digital scale, 9069 PK3R, Tonbridge, UK) and height to the nearest 0.1 cm, using a Stadiometer (Holtain, Crymych, UK). Their waist and hip circumferences were measured using a standard non-stretchable tape at the narrowest point between the iliac crest and ribcage (waist) and at the level of the greater trochanter (hip). Body composition was assessed using DXA (Model DPXMD 7254, Lunar Corporation, Madison, WI). The mass of lean soft tissue, fat, and bone mineral for both the whole body and specific regions were measured. The defined central adiposity included fat (kg) in the trunk, including the android region. The trunk region included the neck, chest, abdominal, and pelvic areas. Its upper perimeter was the inferior edge of the chin and the lower borders intersected the middle of the femoral necks without touching the brim of the pelvis. The android region was the area between the ribs and the pelvis and was totally enclosed by the trunk region. Appendicular muscle index (AMI) an indicator of sarcopenia status was derived using appendicular muscle mass (AMM) which is equivalent to the sum of lean soft tissue in both the right and left arms and legs (19). Equation used for AMI was, AMI = AMM/Ht^2^ (20).

### Muscle Strength and Contractile Quality

Skeletal muscle strength of the knee extensors (quadriceps) of the dominant leg was assessed using an isokinetic dynamometer (Kin Com AP1, Chattanooga Group, Tennessee, USA). All participants underwent familiarization session before the actual protocol. For majority of the participants, familiarization was performed on the previous day of the experiment. If participants could not come on 2 days, we performed the familiarization session on the experimental day. However, adequate rest period (1–2 h) was given before the actual skeletal muscle strength assessment. Participants were instructed to perform a standardized 5-min warm-up indoor brisk walk, following which they were seated in the dynamometer in an upright position. Stabilization straps were placed across the chest and the hip to prevent the involvement of upper limb and trunk muscles during the test. The thigh was stabilized by placing support over the distal third of the thigh. The axis of dynamometer was aligned with the axis of rotation of knee (lateral condyle) and the distal support pad was placed proximal to the malleoli. The lever arm between the center of rotation of the dynamometer and the point of application of force-length was recorded. Maximal isometric torque (0 degree/s) was measured with the lever arm locked at the position of 30 degrees of knee joint extension from the knee flexed (90 degree) position. Peak isokinetic strength for knee extensor was assessed at 3 angular velocities 60, 120, 180 degree/s. A slow angular velocity of 60 degrees/s was used as the main dynamic measure for relation to the metabolic data as this angular velocity allows sufficient time for force development and complete muscle activation to have occurred at the point of the measurement (21). The best of three maximal voluntary contractions for knee extension was used for the analysis. For majority of the participant's right leg was used for the assessment, however if there was any history of pain or injury to the right leg, left leg was used for the assessment. The corresponding lower limb muscle mass (LMM) was considered for the contractile quality calculation. Subjects were provided with a rest interval of 2–3 min between each contraction and 5 min rest between each velocity. Muscle contractile quality was derived by dividing the peak torque (Nm) at each angular velocity by the appropriate lower limb muscle mass (Nm/kg) (22).

### Statistical Analyses

Descriptive statistics are reported as mean and SD/SE for normally distributed continuous variables, else median with 25 and 75th percentiles, and number and percentage for the categorical variables. The comparison of specific characteristics between the three groups was performed using analysis of variance for normally distributed data. *Post-hoc* multiple comparisons were performed using the Bonferroni correction. For the non-normally distributed data, the Kruskal–Wallis test was used to compare between the three groups and the Mann–Whitney *U*-test for two group comparisons. Age-adjusted analyses for between-group differences in muscle strength and contractile quality at various angular velocities were performed using ANCOVA. Multivariate linear regression analysis was performed to find the association between muscle strength and contractile quality for isokinetic strength (60 degree/s) in T2D, PD compared to controls, adjusted for age, percent fat and PAL. The relationships between LMM, strength and contractile quality with FBG, HbA1c, HOMA-IR were assessed using correlation coefficient analyses. Partial correlations were adjusted for age. In all analyses, *P* < 5% was considered statistically significant. All analyses were performed using SPSS version 24.0.

## Results

Among 224 participants enrolled in the study, 24.5, 55.8, and 19.6% belonged to the T2D, PD and healthy control groups, respectively. The average self-reported duration of T2D was 3.0 ± 1.5 years. Overall, the mean age of the study subjects was 39.2 ± 10.8 years. Descriptive statistics for the characteristics of study subjects by study groups are presented in Table 1. The mean age of the study subjects was significantly different across the study groups with controls being the youngest.

**Table 1:** Descriptive statistics of the study groups.

**Variable**	**Diabetes (*n* = 55)**	**Pre-diabetes (*n* = 125)**	**Control (*n* = 44)**
Age (yr)	48.0 ± 8.7^a^, ^c^	37.6 ± 9.3^b^	32.6 ± 11.4
Height (m)	1.68 ± 0.07	1.68 ± 0.06	1.66 ± 0.06
Weight (kg)	68.5 ± 11.0^c^	72.5 ± 9.2	70.6 ± 9.3
Body mass index (kg/m^2^)	24.7 ± 3.9	25.5 ± 2.7	24.8 ± 3.1
Percent fat (%)	29.3 ± 6.6	31.8 ± 5.7	29.2 ± 7.5
Android fat (kg)	2.2 ± 0.8	2.3 ± 0.8^b^	2.0 ± 0.8
Total lean mass (kg)	46.1 ± 4.8	46.6 ± 4.6	47.1 ± 5.3
Muscle to fat ratio^*^	2.4 (1.9, 2.9)	2.1 (1.8, 2.5)^b^	2.3 (1.9, 2.8)
AMI index (kg/m^2^)	9.2 ± 6.8	9.9 ± 4.2	10.4 ± 0.9
Waist hip ratio	0.93 ± 0.06^a^, ^c^	0.89 ± 0.06	0.90 ± 0.06
FBG (mg/dl)	167.4 ± 64.8^a^, ^c^	100.3 ± 9.4	90.3 ± 5.7
HbA1c (%)	8.6 ± 2.2^a^, ^c^	5.7 ± 0.4	5.3 ± 0.2
Basal Insulin (μU/ml)^*^	11.9 (6.9, 18.2)	11.3 (7.4, 15.5)	9.8 (6.9, 13.7)
Total cholesterol (mg/dl)	176.6 ± 47.9	183.0 ± 36.6	177.3 ± 44.2
Triglycerides (mg/dl)^*^	169 (115, 242)^a^, ^c^	126 (89, 169)	125 (81, 161)
HOMA-IR^*^	1.8 (1.2, 2.8)^c^	1.5 (1.0, 2.1)	1.3 (0.9, 1.8)
HOMA-β^^*^^	55.2 (35.8,83.9)^a^, ^c^	66.5 (48.2, 97.9)	78.5 (55.9, 112.7)
Physical activity level	1.6 ± 0.16^c^	1.5 ± 0.13	1.5 ± 0.21

a *Indicates diabetic group significantly different from control group*.

b *Indicates pre-diabetes group significantly different from control group*.

c *Indicates diabetic group significantly different from pre-diabetes group*.

*kg, Kilogram; m, meter; AMI, Appendicular muscle index; FBG, Fasting blood glucose; mg, milligram; dl, decilitre; HOMA, Homeostatic model assessment; IR, insulin resistance; β, Beta cell function*.

* *Reported as Mean ± SD, median (25 and 75th percentile)*.

There was no significant difference in the mean BMI, percent fat and total lean mass between the groups (Table 1). The mean android fat and muscle to fat ratio were significantly different between PD and control groups with no significant difference between T2D and prediabetes groups. The AMI was similar across the study groups. The reported levels of physical activity were significantly different between the PD and T2D groups. The AMI for T2D and PD study groups were not ≤ 2 SD of the young control mean values.

Basal plasma insulin levels were not significantly different between the groups. Mean FBG, HbA1c, and HOMA -IR were significantly higher in T2D compared to PD and control groups, with no significant difference between PD and control groups. Mean HOMA β was significantly lower in T2D compared to PD and control groups.

**Figure 1:**
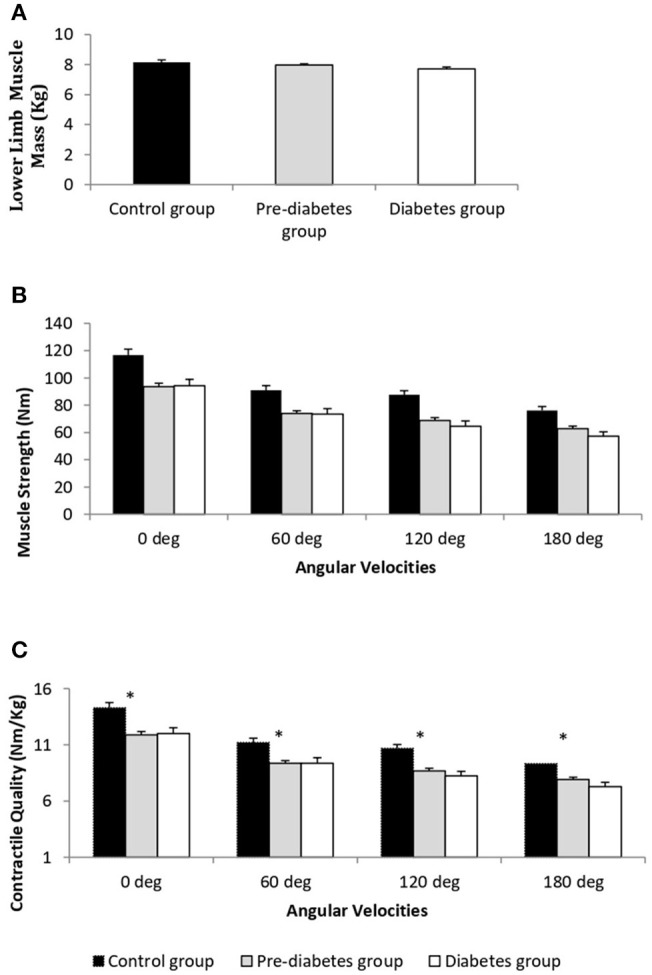
Comparison of lower limb muscle mass (kg) **(A)**, absolute muscle strength (Torque, Nm) **(B)**, and muscle contractile quality (Nm/kg limb muscle mass) **(C)** in the diabetes (*n* = 55), pre-diabetes (*n* = 125), and control (*n* = 44) groups. ^*^T2DM and PD groups are significantly different from control group *p* < 0.01; age adjusted data represented as mean ± SE.

The comparison of LMM between the study groups is represented in Figure 1A. Mean LMM was not significantly different between the groups (*p* = 0.07). The comparison of muscle strength and contractile quality between the study groups at the different angular velocities is shown in Figures 1B,C, respectively. There was no significant association between age with muscle strength, and contractile quality (ρ = −0.11, *P* = 0.11; ρ = −0.12, *P* = 0.08). However, age was significantly different between the study groups. When an age-adjusted analysis was performed the data still showed significant differences in absolute muscle strength (torque) and strength normalized for muscle mass (contractie quality) between the study groups (*p* ≤ 0.01) across all angular velocities. Age-adjusted results revealed that both maximal muscle strength and contractile quality were significantly lower in T2D and PD groups compared to the control group (*p* ≤ 0.01). However, there were no significant differences between the T2D and PD groups in any of the functional measurements. Muscle strength and contractile quality derived from isometric (0 degree) and isokinetic (60 degree) were compared between the three groups using multiple linear regression adjusting for different covariates (Table 2). After adjusting for age, percent fat and PAL as co-variates, muscle strength (β = −24.6, 95% CI −35.9 to −13.2 for isometric; β = −17.2, 95% CI −26.5 to −8.0 for isokinetic) and contractile quality (β = −2.75; 95% CI −4.02 to −1.47 for isometric; β = −2.04; 95% CI −3.12 to −0.95; for isokinetic) were significantly lower in PD compared to controls with no significant difference between PD and T2D or Control and T2D. There was a significant correlation between skeletal muscle strength and contractile quality with the duration of type 2 diabetes (ρ = −0.33, *P* = 0.04; ρ = −0.34, *P* = 0.03). After adjusting the same with age, body fat and PAL in regression analysis the significance disappears (β = −4.18, *p* = 0.06; β = −0.10, *p* = 0.17).

**Table 2:** Multivariate regression models for muscle mass, strength, and contractile quality across the study groups.

	**Leg lean mass**		**Muscle strength**				**Muscle contractile quality**		
	**β**	**95% C.I**.	***P*-value**	**β**	**95% C.I**.	***P*-value**	**β**	**95% C.I**.	***P*-value**
**Control vs. PD**
Model	−0.09	−0.44 to 0.25	0.60	−17.2	−26.5 to −8.00	<0.001	−2.04	−3.12 to −0.95	<0.001
**PD vs. T2D**
Model	−0.18	−0.56 to 0.20	0.35	−0.59	−9.88 to 8.69	0.90	0.13	−0.97 to 0.23	0.82
**Control vs. T2D**
Model	−0.37	−0.91 to 0.18	0.18	−9.49	−23.6 to 4.64	0.18	−0.62	−2.30 to −1.6	0.46

*Adjusted for age, percent fat, PAL; CI, confidence interval; PD, prediabetes; T2D, Type 2 Diabetes*.

**Figure 2:**
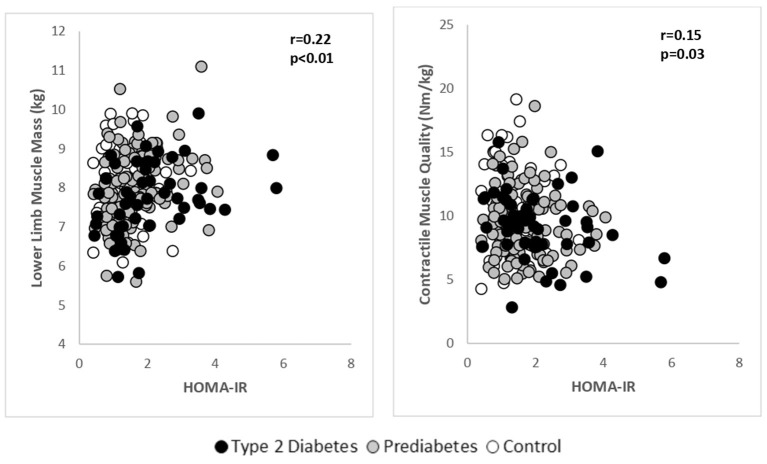
Age adjusted relationships between lower limb muscle mass and contractile muscle quality with HOMA-IR in the pooled study groups.

A pooled analysis was performed to explore the relationship between LMM, muscle strength and contractile quality with HOMA-IR (Figure 2). After adjusting for age, there was a significant positive association between LMM and HOMA-IR (*r* = 0.22, *p* < 0.01). Skeletal muscle strength was not associated with HOMA-IR, however, there was a significant, inverse relationship between muscle contractile quality (Isokinetic 60 degree) and HOMA-IR (*r* = −0.15, *p* = 0.03). It was noted that for every unit increase in HOMA-IR, muscle contractile quality decreased by 0.54 Nm/kg (β = −0.54; 95% C.I. −0.97 to −0.09). Age-adjusted associations between FBG, HbA1c with LMM, muscle strength and contractile quality were not significant.

## Discussion

This data is the first to examine skeletal muscle mass and function in major limb muscles in regard to metabolic disease in an Indian population. The data from the present study shows that quadriceps function when measured both in absolute (Nm) and relative (Nm/kg muscle mass) terms, and for both isometric and dynamic contractions, was similar between individuals with PD and T2D. However, the contractile function was lower in both of these groups compared with healthy controls, even when allowing for the younger age of the controls. Interestingly however, muscle mass (LMM) did not differ between the three study groups when corrected for age. The study also revealed a significant positive association between insulin resistance (HOMA-IR) and muscle mass for pooled data, whilst muscle contractile quality was inversely and significantly associated with insulin resistance.

A loss of muscle mass and function has been reported among T2D individuals particularly in the lower extremities compared to healthy individuals (23). The majority of these reports come from European (24) or American (25) or East Asian elderly population (26) with T2D, in whom age-related changes cannot be separated from the changes due to their diabetes status (27, 28). One possible explanation for the lower force per unit mass in the PD and T2D individuals in the present study concerns the level of neural drive to the muscle during the voluntary contractions. The assumption was that this would be similar in the three groups, but it cannot be excluded that PD and T2D individuals were less able to activate their knee extensor muscles during contractions. For activations to be determined, the twitch interpolation technique (29) would need to be utilized, which was not possible in the present study.

The proposed mechanisms by which T2D/PD could by themselves induce changes in skeletal muscle, beyond aging, could be the independent effect of insulin resistance on mitochondrial dysfunction, protein degradation, and autophagy pathways in the skeletal muscle (30–32). One of the potential mechanisms by which insulin resistance could induce mitochondrial dysfunction and negatively impacting muscle mass and function could be lipid accumulation or the lipotoxicity hypothesis. Excessive lipid accumulation due to defective muscle lipid oxidation could cause impaired mitochondrial number or function (reduced oxidative enzyme capacity) (33). However, Nair et al. demonstrated that Indians irrespective of their diabetes status had higher OXPHOS capacity than Northern European Americans indicating a dissociation between mitochondrial dysfunction and insulin resistance (34). However, this study was performed on a small sample of migrant Indians in the USA. With environmental factors and lifestyle habits differing across native compared to migrant Indians, extrapolation of the data needs further exploration.

There was a significant positive association between lower limb muscle mass and insulin resistance in the present study. This finding has not been reported earlier in Asian Indians. The majority of studies have demonstrated an inverse association between insulin resistance and muscle mass. For instance, the third National Health and Nutrition Examination Survey demonstrated that relative muscle mass, as estimated by bioelectrical impedance, was inversely associated with insulin resistance and PD (35). Similarly, a 10-year longitudinal study demonstrated that there was an independent inverse association between insulin resistance and muscle mass among middle-aged and older healthy Americans of Japanese origin (36). However, the Baltimore Longitudinal Study of Aging (2003–2011) reported hyperglycemia was not related to decreased skeletal muscle mass over time among multiracial Americans (37). The fact that muscle mass was related to insulin resistance positively in the current study even after adjusting for age needs further exploration, but maybe explained, in part, by inter- and intramyocellular lipid accumulation, contributing to the muscle bulk. A recently published analysis of muscle biopsy samples from our group showed evidence of excessive intramyocellular fat among Asian Indians with PD compared to healthy controls (6).

Contractile quality was shown to be inversely associated with HOMA-IR in the present study. This finding was similar to The Health ABC elderly cohort (70–79 years), with muscle mass and contractile quality demonstrating similar association, but not with strength (38). The current study population being younger with an average age of 39 years and included PD and T2D compared to the elderly population from the Health ABC elderly cohort. With every unit increase in HOMA-IR, there was a 0.54 Nm/kg decrement in contractile quality in the present study. In a similar study performed on Japanese population with Type 2 Diabetes, it was noted that knee extensor contractile quality decreased by 0.40 Nm/kg (body weight) with every unit increase in HOMA IR (39). The Korea National and Health Examination Survey (KNHANES) VI on Type 2 Diabetes population demonstrated that the hand grip strength normalized for body weight demonstrated a decrease of 3.70 kg/kg with every unit increase in HOMA IR (40).The fact that the current study group might have changes in skeletal muscle earlier than other population emphasizes the need for early evaluation of skeletal muscle mass and function including planning of preventive strategies.

Based on a recent systematic review and meta-analysis, the role of insulin on the human skeletal muscle anabolism through increased muscle protein synthesis (MPS) has been shown to be influenced by amino acid delivery (41). The branched chain amino acid leucine is known to play vital role in the MPS (42, 43). However, the leucine requirement in normal, well-nourished Western and Indian men has been determined to be 2.5 times higher than the 1985 FAO/WHO/UNU recommendation. On the contrary, lysine has been shown to be the most limiting in cereal protein and at a much lower concentration with relatively poor digestibility and utilization (44, 45). With majority of Asian Indians consuming a cereal based diet they pose a risk of a deficiency of quality protein, in terms of indispensable amino acid (particularly lysine). This is due to the fact that 60% of their protein are from cereals with relatively low quality and digestibility (46). Whether high-quality protein intake could harness the positive effects of insulin on muscle metabolism needs to be explored particularly among Asian Indians. However, the role of insulin in reducing muscle protein breakdown (MPB) is independent of amino acid availability (37). It is suggested that impaired insulin signaling leading to insulin resistance could have an effect on muscle metabolism (MPS and MPB) and endothelial dysfunction beyond glucose intolerance (47). Further, Mesinovic et al. suggested that there could be a bidirectional link between T2D and skeletal muscle changes, and the existence of one condition may increase the risk of developing the other. Physical inactivity, insulin resistance, inflammation, increased advanced glycation end-products accumulation, increased oxidative stress, and vascular complications can all affect various components of muscle health (muscle mass, strength, and contractile quality) and impaired muscle health could further contribute toward the development and progression of T2D (48). Therefore, it is important to explore, through a multipronged approach, the different mechanisms that could regulate skeletal muscle mass, strength, and contractile quality in Asian Indians.

While muscle function among PD and T2D was reduced in comparison with healthy controls, the PD and T2D groups were comparable in both muscle mass, absolute strength, and contractile quality. We had expected a graded response between the control, PD and T2D study groups as the metabolic disease progressed, even though this was a cross-sectional study. The reasons for this lack of difference between PD and T2D remain to be elucidated, but these findings suggest that in a population where reported prevalence rates of T2D are high, a simple functional evaluation of muscle strength may not only act as an early prognostic tool but also may act as a positive feedback mechanism for participants trying to increase their levels of activity and physical fitness. After adjusting for age, percent fat and PAL there was no significant difference in muscle strength and contractile quality between T2D and control groups. This was surprising and could suggest that there are factors beyond those which been explored as part of the study. The fact that the T2D individuals were on oral hypoglycemic agents (OHA) and which might have had an independent effect on muscle strength and contractile quality cannot be ruled out. With a body of literature suggesting that OHA could cause muscle atrophy and rest demonstrating no effect (49). It is nearly impossible to differentiate the effect of OHA from that of a disease process in diabetes mellitus. With the small sample size and lack of detailed pharmacological profiling available as part of the study, we could only speculate at this stage and could form the basis of future studies.

## Conclusion

Skeletal muscle mass, strength, and contractile quality were similar between PD and T2D Asian Indians, but lower compared to healthy controls, with the findings persisting even after adjusting for the age differences between the groups. This would suggest that the measurement of skeletal muscle function, as well as mass, among Asian Indians is important in order to detect early deterioration in metabolic health. The data also suggest the need to evaluate the role of resistance exercise in improving muscle strength and quality through well-designed clinical studies in Asian Indians.

## Data Availability Statement

The datasets for this manuscript are not publicly available because they are a part of an on-going trial. Requests to access the datasets should be directed to SSa, sucharita.dr@gmail.com.

## Ethics Statement

The studies involving human participants were reviewed and approved by Institutional Review Board, St. John's National Academy of Health Sciences, Bangalore, India. The patients/participants provided their written informed consent to participate in this study.

## Author Contributions

SSa was part of the conception and design, the performance of the measurements and analyses, interpretation of the data, and drafted the manuscript. AK and SH were part of conception and design, interpretation of data, writing of manuscript, and final approval of manuscript. NS was involved in the performance of measurements and analyses. PR involved in screening and recruitment of subjects. SSe was involved in statistical analyses.

### Conflict of Interest

The authors declare that the research was conducted in the absence of any commercial or financial relationships that could be construed as a potential conflict of interest.
